# Longitudinal analysis of radiological parameters after monosegmental lumbar instrumentation and posterior lumbar interbody fusion (PLIF) compared with transforaminal lumbar interbody fusion (TLIF)

**DOI:** 10.1016/j.jor.2025.06.002

**Published:** 2025-06-03

**Authors:** Hassan Awan Malik, Andreas Kiebler, Julia Müller, Thomas Caffard, Timo Zippelius, Luis Ferraris, Heiko Reichel, Balkan Cakir, Tugrul Kocak

**Affiliations:** a Department of Orthopedic Surgery, University of Ulm, Oberer Eselsberg 45, 89081, Ulm, Germany; b Spine Center, Werner-Wicker-Klinik, Im Kreuzfeld 4, 34537, Bad Wildungen, Germany; c Orthopedic Practice Kiebler, Bocksgasse 2, 73525, Schwäbisch Gmünd, Germany; d Department of Orthopedic Surgery, Wertach Kliniken, Wertachstrasse 55, Bobingen, 86399, Germany

**Keywords:** PLIF, TLIF, Lumbar spine

## Abstract

**Introduction:**

Restoring the sagittal balance of the spine has gained significant importance. Still there is little data objectifying the influence of a monosegmental fusion or the implants used.

This is a comparative study that directly contrasts PLIF and TLIF regarding their impact on sagittal balance. In this study 53 patients who received a monosegmental lumbar fusion were followed up.

**Methods:**

53 patients (37 women, 16 men, average age 57.4 years) who received a monosegmental spondylodesis were followed up with an average time of 15.1 months.

To objectify the potential postoperative changes radiographs were made to measure the index segments lordosis as well as the lumbar lordosis overall. A further subdivision was made according to the operated functional spinal segment treated and cage used.

**Results:**

Overall no significant changes in total or segmental lordosis were found. Statistically significant changes were measurable on immediately postoperative radiographs and declined in time, while lumbar lordosis decreased, segmental lordosis increased. Segmental lordosis was consistent. Comparing patients operated in PLIF and TLIF technique the overall lordosis showed the same patterns. Segmental lordosis was increased postoperatively (p = 0.0162). Followed by a significant loss (p = 0.0405). The TM 500 PLIF Cage showed a significantly improved lumbar lordosis over the course compared with postoperative values, but not in comparison to the preoperative values.

**Conclusion:**

We were unable to find significant difference of the sagittal profile after a monosegmental instrumentation and fusion in the lumbar spine. However, the PLIF procedure seems to be superior with regards of lordosis restoration. The L4/5 segment also seems to have a greater potential for correction the L5/S1 segment in the long term.

## 1 Introduction

Back pain ranks amongst Germany's major health problems. Both socially and economically, it poses an enormous challenge. Conservative therapy doesn't always provide sufficient relief for the patient's discomfort, thus only leaving surgical intervention as a long term treatment option. The number of spinal surgeries has been steadily increasing over the years, attributed in part to advancements in spinal implants. Depending on the underlying pathology, various methods exist for surgically treating degenerative spinal conditions. These include both motion-preserving and fusion techniques. Alongside nucleotomies, these surgeries are the most significant operative interventions in spinal surgery.

Interbody fusions are being carried out in increasing numbers. No other orthopedic intervention has become this important in recent years.^7^ The reason for this trend is the development of intervertebral implants, which have increasingly replaced autologous bone in order to achieve lumbar fusion. This shift is also due to the various complications associated with the use of autologous bone^4^^,^^5^ and the increasingly successful outcomes from using interbody cages for spinal fusion.^6^^,^^12^^,^^14^^,^^19^^,^^26^^,^^29^^,^^30^ To place these cages correctly, different surgical techniques have been developed for interbody fusion. The best known techniques are anterior, posterior, and transforaminal lumbar intervertebral fusion. Many studies have examined the results of these different techniques, but there is rarely literature comparing the PLIF and TLIF techniques in terms of sagittal balance.

Depending on the underlying pathology, there are different methods for the surgical treatment of degenerative spinal diseases. The mentioned fusion techniques, along with nucleotomies, represent the most important surgical interventions on the spine.^30^ There are various indications for an interbody fusion. The best outcomes in intervertebral fusions are achieved in spondylolistheses under a 40 % slip.^6^^,^^12^^,^^21^^,^^27^^,^^29^^,^^31^ Other indications for intervertebral fusions include sciatica due to degeneration or previous disc herniations, as well as disc herniations requiring nerve root decompression with neuroforaminal enlargement and fusion.^8^^,^^18^ However, the results cannot be compared to those of spondylolistheses.

The surgical intervention aims to achieve fusion with the goal of eliminating existing instabilities.^24^ To achieve rapid fusion with the lowest possible rate of pseudoarthrosis, motion in the operated segment must be maximally reduced.

This is best achieved through the combination of dorsal instrumentation and intercorporal fusion using a cage or an iliac crest bone graft as used in the past.^1^^,^^2^

Different techniques are distinguished based on the type of approach and instrumentation used. Operative approaches can be anterior, posterior, or lateral, each having its own specific advantages and disadvantages.

As a placeholder in the intervertebral space, autologous bone was initially inserted after surgically removing the disc. However, significant drawbacks such as complications during extraction, pseudoarthrosis, dislocations, and collapses became evident over time. Therefore, implants were developed with the goal of providing high primary stability, low tendency for dislocation, restoration and maintenance of the intervertebral space, and restoration of the natural lumbar lordosis. Additionally, these cages were intended to possess osteoinductive and osteoconductive properties to expedite fusion.^8^^,^^35^ Different types of cages have been developed to date with varying properties, including 'open box,' 'horizontal cylinder,' and 'vertical ring' cages.

The development of modern implants has significantly expanded the surgical treatment options for spinal pathologies. Furthermore, there has been an increasing focus not only on the local structural pathologies but also on spinal statics.

Various parameters are considered when evaluating the sagittal profile of the spine. These parameters include sacral slope, total lordosis, C7 plumb line and pelvic parameters, such as pelvic tilt and pelvic incidence.^10^

Measuring the above mentioned radiographic parameters in the spine and pelvis guides therapy planning, aiming not only for treatment of the local structural pathologic findings but also for the restoration of sagittal balance overall.^10^^,^^20^ Specifically the restoration of segmental lordosis in monosegmental spondylodesis is of great importance. Failure to ensure this can lead to compensatory mechanisms in the untreated functional spinal unit,^28^ ultimately leading to premature adjacent segment disease.^17^ Furthermore, a correlation has been established between back pain and loss of lordosis following spondylodesis with PLIF.^11^

Consequently, this study was carried out to determine whether differences in the total lordosis of the lumbar spine or the segmental lordosis could be identified in patients receiving a monosegmental dorsal instrumentation and interbody fusion, depending on factors such as the surgical approach (TLIF vs. PLIF), the cages used or the functional units addressed.

## 2 Materials and methods

An ethics committees approval was obtained prior to the study. For this study we selected patients who were treated with a monosegmental dorsal instrumented spondylodesis and interbody fusion using the TLIF- or PLIF-technique between April 2007 and January 2013 at the University Hospital of Ulm, Germany. Surgical indications for the patients included spondylolisthesis (degenerative/isthmic), advanced segment degeneration with secondary spinal canal stenosis or post-nucleotomy syndrome. Patients were included if preoperative, immediate postoperative, and follow-up sagittal radiographic images of the entire lumbar spine existed.

The cages that were used were 9 times a TM 500 Cage by Zimmer (PLIF procedure), 13 times a Capstone-Cage by Medtronic (TLIF procedure), 19 times a Pezo-P Cage by Ulrich (PLIF procedure) and 12 times a Pezo T-Cage by Ulrich (TLIF procedure) (Fig. 1a, Fig. 1b, Fig. 1ca–c).

**Fig. 1a:**
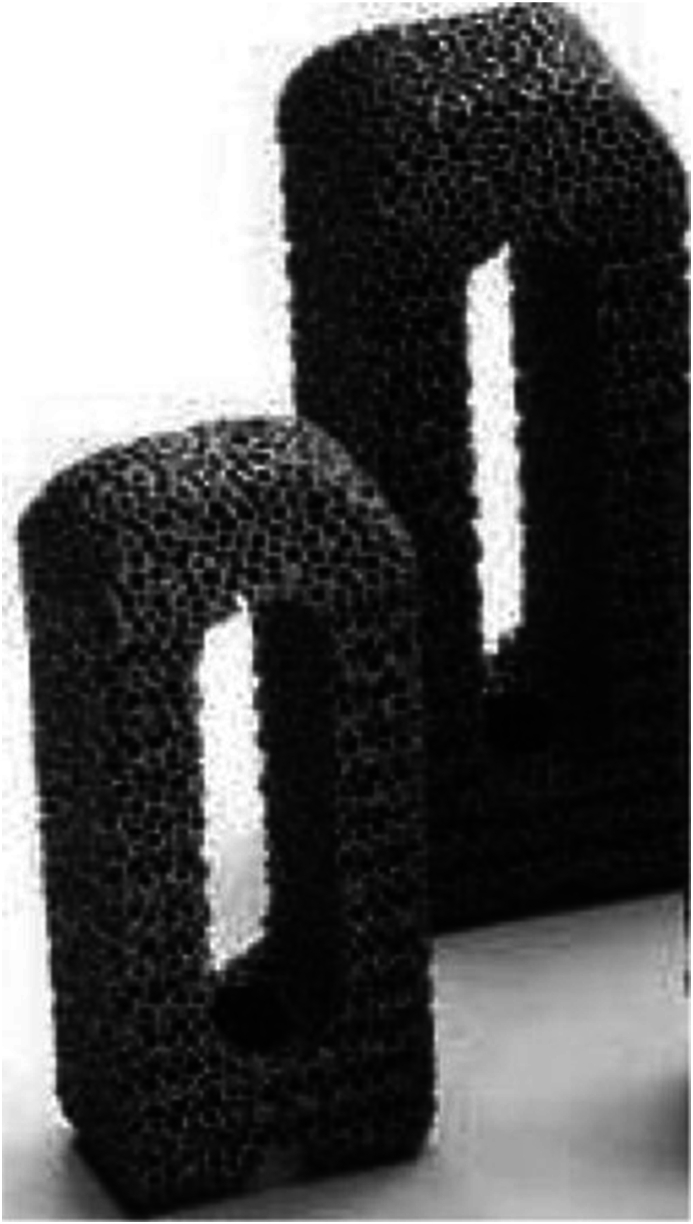
TM 500 cage by zimmer (courtesy of zimmer biomet)^36^.

**Fig. 1b:**
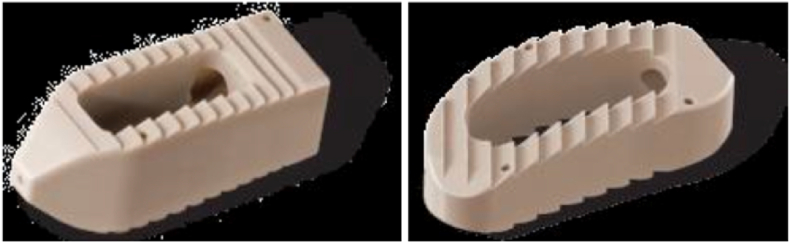
Pezo-P (left image) and Pezo-T (right image) by Ulrich (Courtesy of Ulrich GmbH & Co. KG)^32^.

**Fig. 1c:**
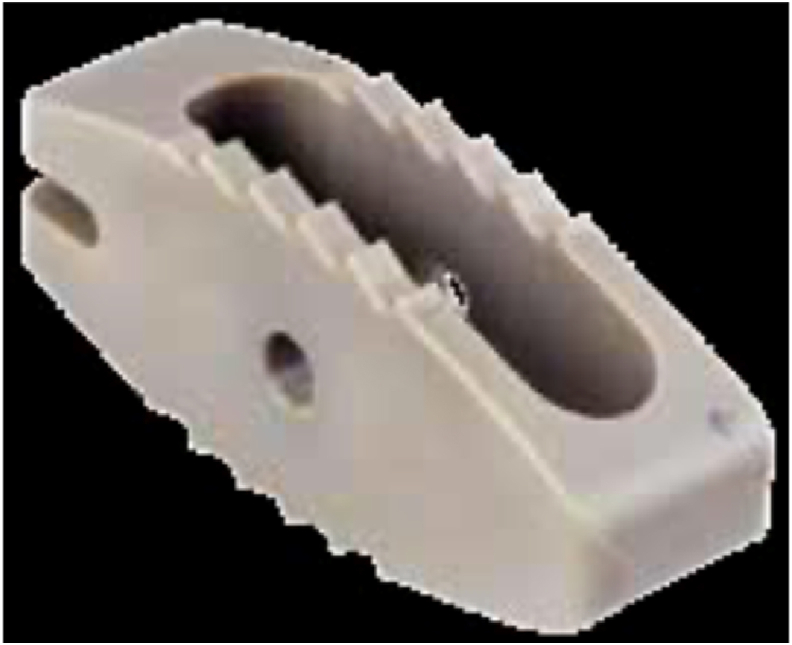
Capstone Cage by Medtronic (Courtesy of Medtronic Deutschland GmbH. Capstone® Cage Family includes a technology developed by Gary K. Michelson, MD)^3^.

X-ray images of the lumbar spine in two planes (lateral and anterior-posterior) were obtained for all patients both preoperatively and postoperatively. As part of the follow-up, preoperative, immediately postoperative, and the latest follow-up images were measured.

In the conducted X-ray examinations, both the total lordosis and the segmental lordosis in the operated segment were determined. Segmental lordosis was determined using the Cobb method. This involved measuring the angle between a line drawn on the superior endplate of the cranial vertebral body and a line drawn on the inferior endplate of the caudal vertebral body. The same procedure was applied in the L5/S1 segment, where the inferior endplate of S1 was determined by adjusting the contrast and brightness values.

**Fig. 2:**
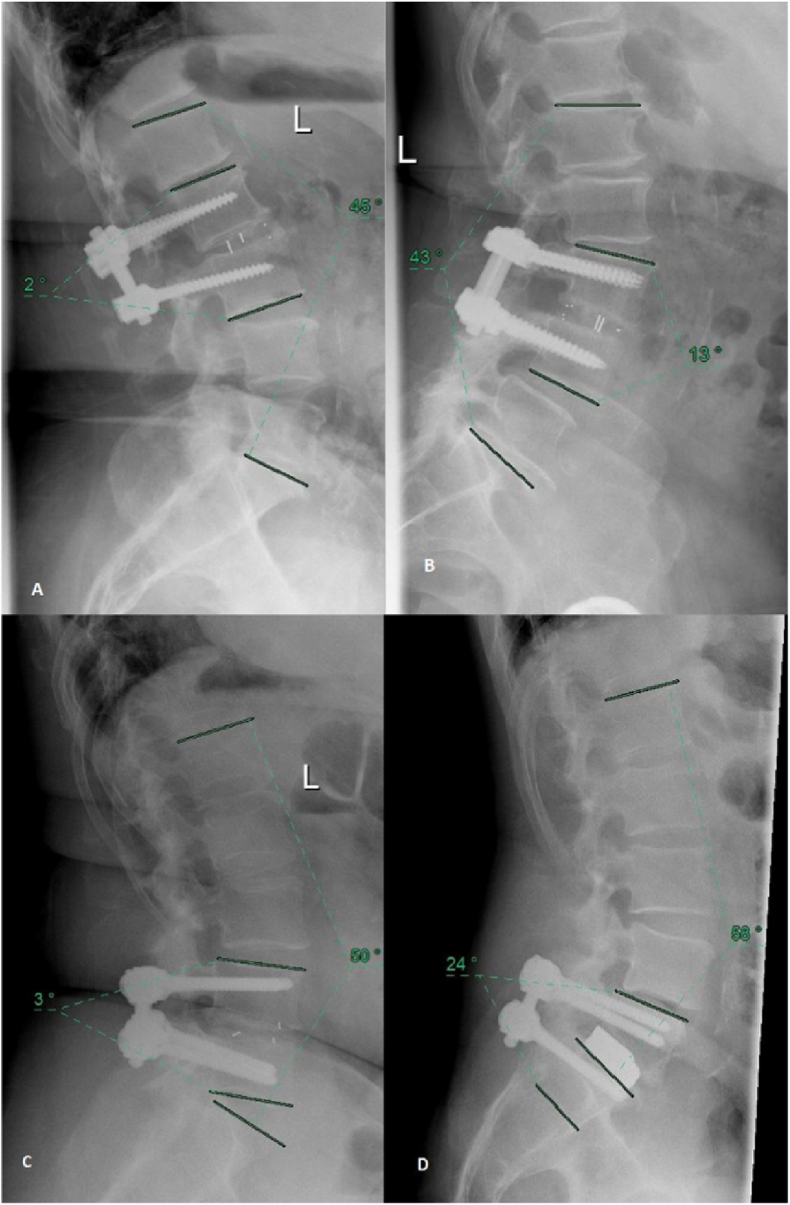
Examples of measured segment and total lordosis in the fused segment L2/3 with a Pezo-T Cage (A), L3/4 with a Pezo-P Cage (B), L4/5 with a Capstone-PEEK Cage (C), and L5/S1 with a TM 500 Cage (D).

Measurement of the total lordosis was performed based on the angle between a line drawn on the superior endplate of L1 and a line drawn on the superior endplate of S1 (Fig. 2).

To determine the measurement accuracy, the total lordosis of all 53 patients was determined twice preoperatively, postoperatively and during follow-ups. At least three months elapsed between the examiner's measurements.

For statistical analysis, the Wilcoxon rank-sum test was employed, which, unlike the Student's t-test, does not require data to follow a normal distribution.^14^ A significance level of p-value 0.05 was set.

## 3 Results

The study group comprised 53 patients, including 37 females and 16 males, with an average age of 57.4 ± 16.2 years. The average age for females was 59 ± 17.3 years and for males 53.8 ± 12.9 years. 25 patients (9 males, 16 females) underwent surgery using the TLIF technique, while 28 patients (7 males, 21 females) underwent surgery using the PLIF technique. Among those operated using the PLIF technique, 24 patients had a preoperative diagnosis of spondylolisthesis (degenerative/isthmic), and 4 patients had a secondary spinal canal stenosis due to advanced segment degeneration. In the TLIF-operated group, 14 patients were operated due to spondylolisthesis (degenerative/isthmic), 6 patients due to secondary spinal canal stenosis with advanced segment degeneration, and 5 patients due to post-nucleotomy syndrome.

The average radiological follow-up interval for the entire collective was 15.1 months. It was 17 months for the PLIF group and 12.9 months for the TLIF group. Among the 28 patients operated using the PLIF technique, 2 patients were operated at the L3/4 level, 12 patients at the L4/5 level, and 14 patients at the L5/S1 level. In the TLIF collective, 1 patient was operated at the L2/3 level, 4 patients at the L3/4 level, 14 patients at the L4/5 level, and 6 patients at the L5/S1 level.

Among the patients operated using the PLIF technique, 19 patients were provided with the Pezo-P Cage by Ulrich (2 patients at L3/4, 9 patients at L4/5, and 8 patients at L5/S1), and 9 patients received the TM 500 Cage by Zimmer (3 patients at L4/5, 6 patients at L5/S1). In the TLIF group, 12 patients were provided with the Pezo-T Cage by Ulrich (1 patient at L2/3, 2 patients at L3/4, 5 patients at L4/5, and 4 patients at L5/S1), and 13 patients received the Capstone Cage by Medtronic (2 patients at L3/4, 9 patients at L4/5, and 2 patients at L5/S1).

**Fig. 3:**
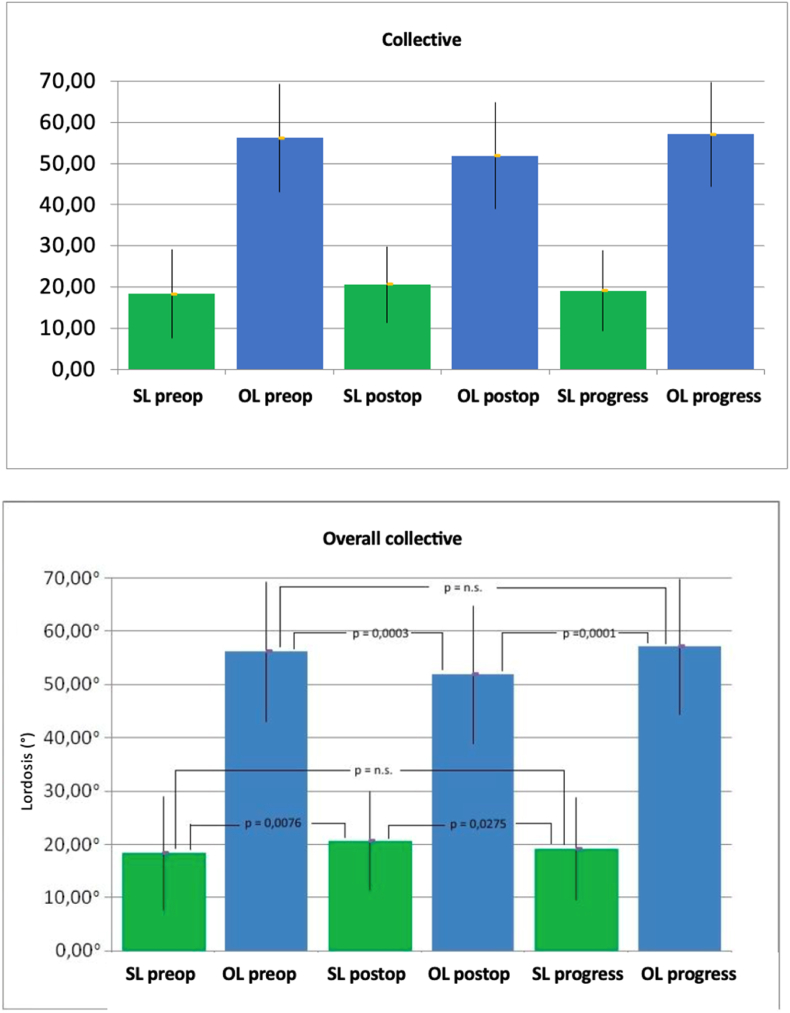
Mean values and standard deviation of the segmental lordosis (SL) and total lordosis (GL) both preoperatively (pre), postoperatively (post) and over the course in degrees (°) in the overall collective (Dept. of Orthopedics of Ulm University Hospital 2007–2013). (p = significance level, n.s. = not significant).

When considering the entire collective, there were no significant changes observed in either total lordosis (preoperative 56.18° ± 13.11°, follow-up 57.13° ± 12.73°; p = 0.3582) or segmental lordosis (preoperative 18.34° ± 10.74°, follow-up 10.15° ± 9.71°, p = 0.3255), except immediately postoperative changes, which were not sustained. While lumbar lordosis decreased to 51.84° ± 12.98° (p = 0.0003) directly postoperatively, there was an increase in segmental lordosis to 20.67° ± 9.27° (p = 0.0076) (Fig. 3).

**Fig. 4:**
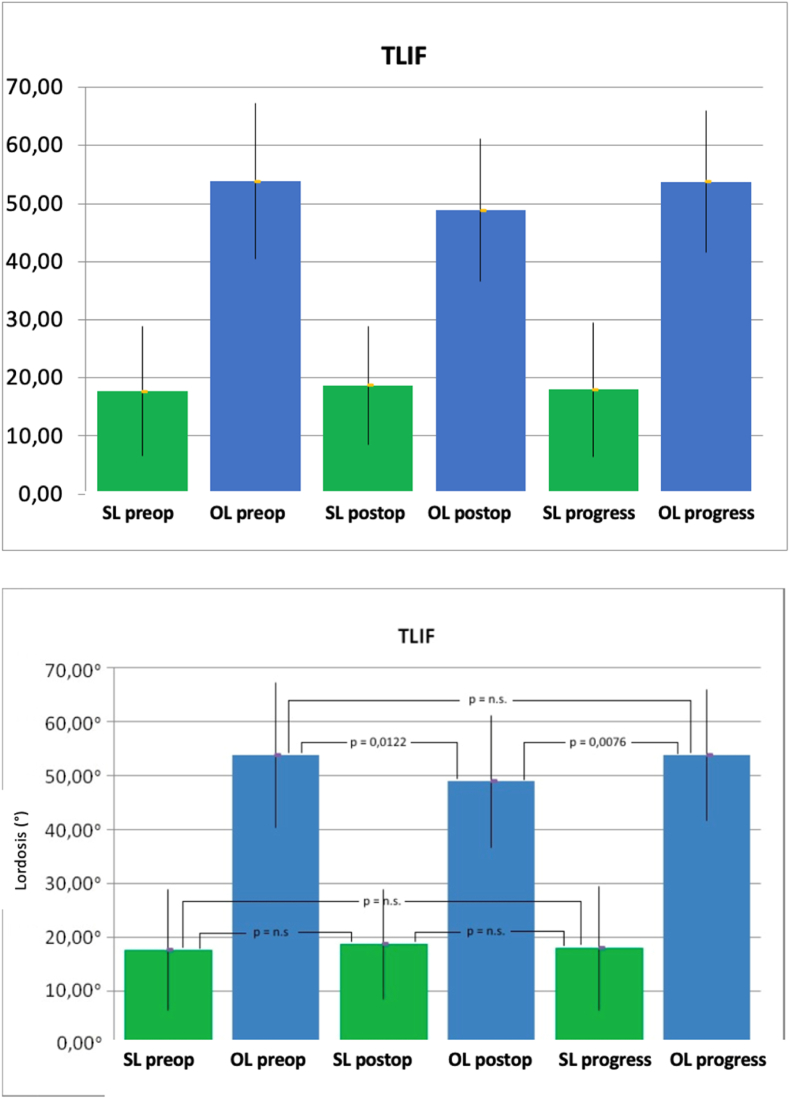
Mean values and standard deviation of the segmental lordosis (SL) and total lordosis (GL) both preoperatively (pre), postoperatively (post) and over the course in degrees (°) of patients treated using TLIF technique (Dept. of Orthopedics of Ulm University Hospital 2007–2013). (p = significance level, n.s. = not significant, TLIF: transforaminal lumbar interbody fusion).

In patients operated using the TLIF technique, there was a postoperative significant loss in total lordosis from 53.82° ± 13.41°–48.87° ± 12.23° (p = 0.0122) and a significant increase during the follow-up to 53.73° ± 12.19° (p = 0.0076). Ultimately, this indicated no difference between the preoperative values and the values of total lordosis during the follow-up (p = 0.9485). There were no significant differences observed in segmental lordosis at any point (comparison of preoperative with follow-up, p = 0.7734) (Fig. 4).

**Fig. 5:**
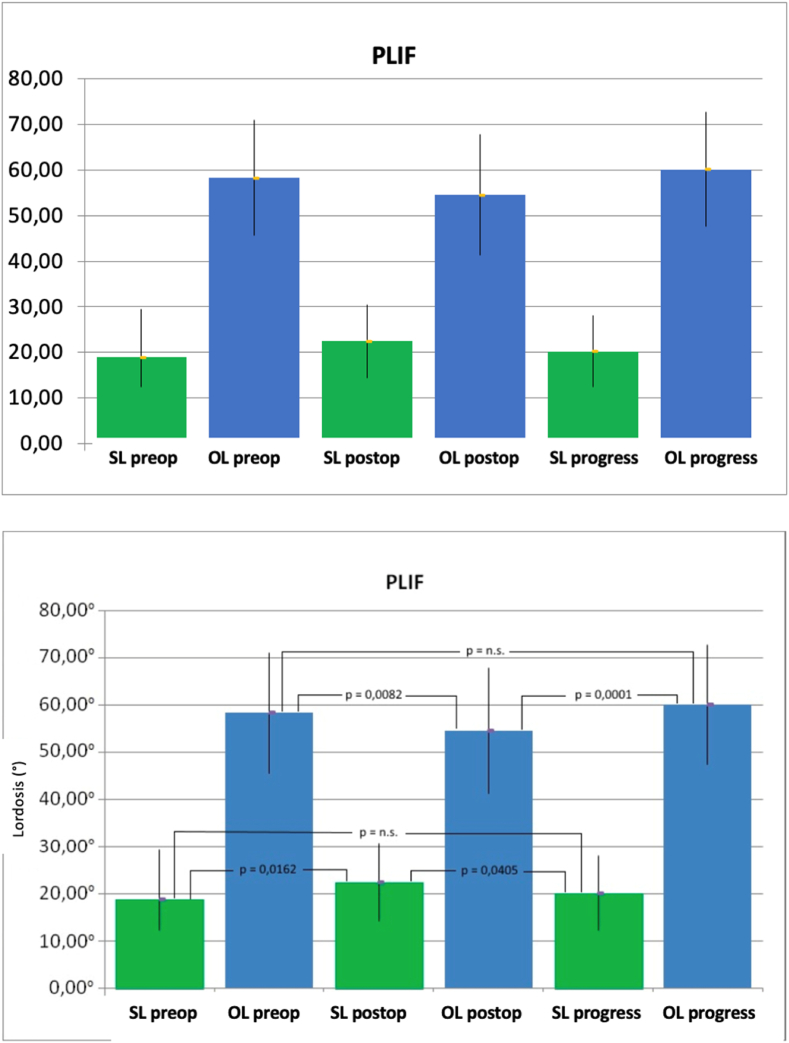
Mean values and standard deviation of the segmental lordosis (SL) and total lordosis (GL) both preoperatively (pre), postoperatively (post) and over the course in degrees (°) of patients treated using PLIF technique (Dept. of Orthopedics of Ulm University Hospital 2007–2013). (p = significance level, n.s. = not significant, PLIF: posterior lumbar interbody fusion).

In patients operated using the PLIF technique, similar patterns were observed as in those operated using the TLIF technique. After a significant postoperative loss of total lordosis from 58.29° ± 12.70°–54.50° ± 13.26° (p = 0.0082), there was a significant gain in lordosis to 60.17° (p = 0.0001). Ultimately, compared to the preoperative total lordosis, there was a non-significant increase during the follow-up (p = 0.2265). For segmental lordosis, there was a significant increase postoperatively from 18.93° ± 10.49°–22.43° ± 8.12° (p = 0.0162). Subsequently, there was a significant decrease to 20.20° ± 7.83° (p = 0.0405). The measured increase between preoperative lordosis in the operated segment and during the follow-up was found to be non-significant (p = 0.3216) (Fig. 5).

**Fig. 6:**
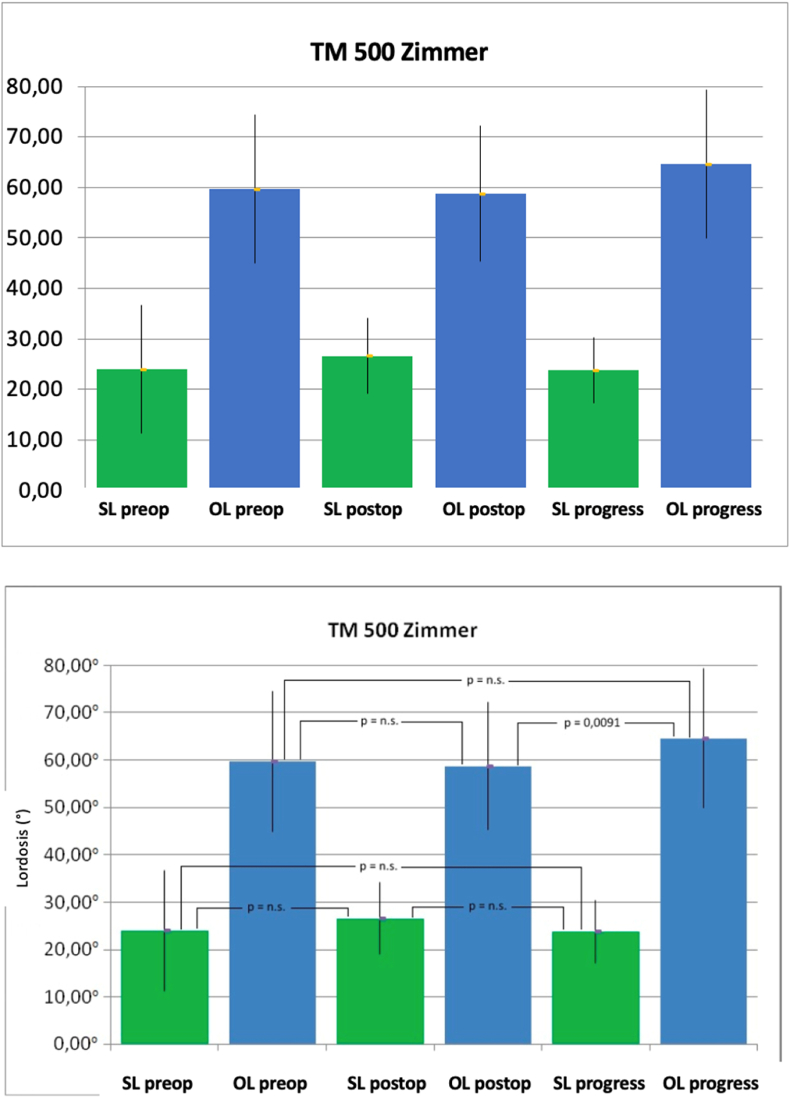
Mean values and standard deviation of the segmental lordosis (SL) and total lordosis (GL) both preoperatively (pre), postoperatively (post) and over the course in degrees (°) of patients treated with TM 500 Cage from Zimmer (Dept. of Orthopedics of Ulm University Hospital 2007–2013). (p = significance level, n.s. = not significant).

**Fig. 7:**
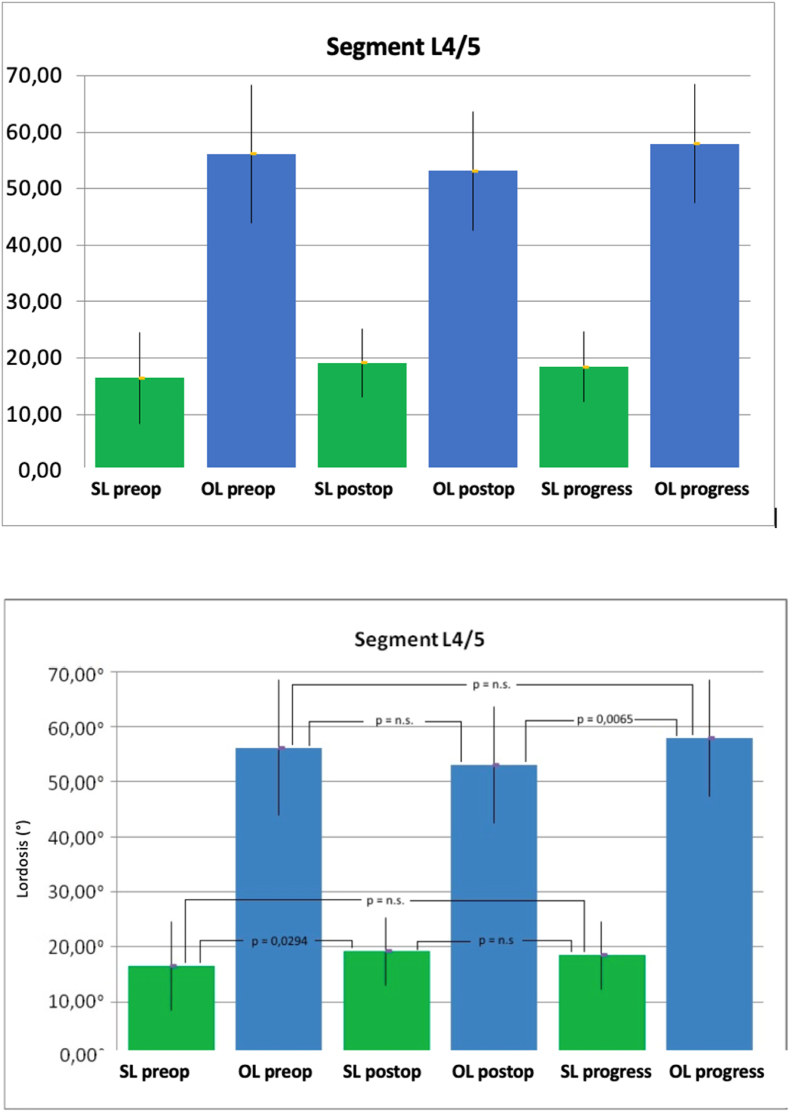
Mean values and standard deviation of the segmental lordosis (SL) and total lordosis (GL) both preoperatively (pre), postoperatively (post) and over the course in degrees (°) of patients treated at L4/5 (Dept. of Orthopedics of Ulm University Hospital 2007–2013). (p = significance level, n.s. = not significant, L: lumbar vertebrae).

The behavior observed in the used cages mirrored that of the corresponding surgical technique. However, among patients with the TM 500 PLIF Cage from Zimmer, there was a significantly improved lumbar lordosis during the follow-up period (64.58° ± 14.72°) compared to the postoperative values (58.70° ± 13.43°; p = 0.0091). This improvement, however, was not significant compared to the preoperative values (59.68° ± 14.76°; p = 0.2373) (Fig. 6). When assessing the operated segment, the L4/5 segment showed a tendency towards the best results over the long term, although this was not statistically significant (Fig. 7).

## 4 Discussion

In conclusion, there was no significant correction observed in the sagittal profile with monosegmental treatment regarding segmental and total lordosis in this study. However, the use of the PLIF technique appears to be potentially superior to the TLIF technique. Additionally, the L4/5 segment seems to have greater long-term correction potential compared to the L5/S1 segment, although this did not reach statistical significance.

Regarding the total lordosis in this study, a general postoperative loss is observed, regardless of the surgical technique or the operated segment. Studies have shown that the PLIF and TLIF techniques do not exhibit advantages over each other in restoring the spinopelvic balance.^38^

Even though this loss is not significant, the tendency is evident in all cases. However, recovery always occurs during the follow-up, resulting in no significant difference between the preoperative and follow-up values. Ultimately, no clear influence on total lordosis can be identified through monosegmental spondylodesis with intercorporal fusion. The cause of postoperative lordosis loss could be attributed to persistent pain from the surgical procedure. Additionally, the surgical approach creates significant trauma in the area of the back extensors. Through the healing process, there might be a normalization of function, which could potentially correct the postoperative loss in total lordosis, making no significant difference between preoperative and postoperative values. Moreover, compensatory mechanisms existing preoperatively might not be as necessary following successful treatment of a pathological segment.

Kong et al.^16^ established a relationship between existing pain and sagittal profile. Patients with a pain reduction <3 on the visual analogue scale (VAS) after surgery exhibited lower lumbar lordosis than those with a pain reduction >3 on the VAS scale. Only patients treated with the TM 500 Cage by Zimmer showed an improvement in total lordosis. However, it's essential to mention that this constitutes the smallest collective with only 9 patients. Larger sample studies would be necessary to confirm these results.

Recent literature presents inconsistent findings concerning the examination of lumbar lordosis. Some studies also reported no increase in total lordosis.^13^^,^^34^ However, there are studies reporting an increase in lumbar lordosis.^9^^,^^16^^,^^23^ This might also be caused by the relationship between pain and lordosis. For instance, Liow et al. showed that patients who underwent monosegmental TLIF surgery at the L4/5 level, resulting in a sacral slope of >30°, accompanied by increased lumbar lordosis, experienced fewer postoperative back pain.^39^ This was attributed to the overall better sagittal balance.^39^

The situation is reversed for segmental lordosis compared to total lordosis. In segmental lordosis, there is a significant increase postoperatively, which cannot be sustained during the follow-up. For patients operated with the TLIF technique, no significant improvement in segmental lordosis was achieved in this study. This applies to both TLIF-Cages used (Pezo-T, Ulrich; Capstone, Medtronic).

A better outcome is observed in patients operated with the PLIF technique. Postoperatively, there is a significant increase in segmental lordosis, indicating a trend towards an increase in segmental lordosis despite a loss of correction during follow-up. Better results were seen with the use of the Pezo-P Cage from Ulrich compared to the TM 500 Cage by Zimmer. However, it's essential to note that the collective, where the TM 500 Cage was used, was the smallest.

A theoretical explanation for this tendency of PLIF's superiority over TLIF could be due to better access to the disc space from both sides in PLIF, possibly resulting in a greater gain in segmental lordosis. In TLIF, the contralateral facet joint could act as a barrier to repositioning. This hypothesis would require verification through biomechanical studies. However, Yson et al.^37^ showed initial indications of this after bilateral TLIF. Additionally, the maximum achievable correction in both techniques is limited by the anterior longitudinal ligament. The relevance of the anterior longitudinal ligament in achievable segmental lordosis was demonstrated by Schmidt et al.^26^ with implants (disc prostheses) introduced ventrally, indicating that complete resection of the anterior longitudinal ligament tends to achieve greater segmental lordosis.

One approach to explaining the loss of correction that occurs during the follow-up in segmental lordosis is the following hypothesis: There might be an unequal distribution of force between the anterior column (with the cages introduced under distraction) and the dorsal instrumentation immediately postoperatively. This could explain a relative loss of segmental lordosis due to partial migration of the cage into the endplates until reaching a balance in load distribution. There are no studies available at present to investigate this issue. However, for the maximally achievable surgical segmental lordosis, the size of the cage used and its positioning is also relevant. In this study all surgeries were performed by one surgeon only. However, there are greater limitations in the PLIF and TLIF techniques due to the surgical access used compared to a cage introduced in ALIF technique.

When comparing the results of segmental lordosis at different levels, it's observed that no correction can be sustained at the L5/S1 level. While there was a slight correction achieved at the L3/4 level, the greatest correction potential was observed at the L4/5 level. Even though the postoperative significant increase in lordosis was not entirely sustained during follow-up, a clear trend can be identified. A potential explanation lies in the fact that the largest portion of lumbar lordosis comes from the lowest 2 segments. Consequently, the physiological segmental lordosis is the largest here. If there is a loss of segmental lordosis due to pathologies, the greatest correction potential arises in this area. However, due to the position of the endplates at the L5/S1 level, a lesser distraction can be achieved through a dorsal approach compared to the L4/5 level.

## CRediT authorship contribution statement

**Hassan Awan Malik:** All authors read and approved the final manuscript. **Andreas Kiebler:** Formal analysis, interpreted and wrote study. **Julia Müller:** collected the patient data and created the statistics and graphs. **Thomas Caffard:** interpreted the statistics and co-wrote the interpretation. **Timo Zippelius:** interpreted the date and analyzed the data regarding the outcome of the implants used. **Luis Ferraris:** interpreted the date and analyzed the data regarding the outcome of the implants used. **Heiko Reichel:** interpreted the date and analyzed the data regarding the outcome of the implants used. **Balkan Cakir:** interpreted the date and analyzed the data regarding the outcome of the implants used. **Tugrul Kocak:** are to be seen as senior authors and pioneered the hypothesis as well as guided the study.

## Ethical statement

The ethics committee of the university clinic of Ulm, Germany approved this study.

## Guardian consent

This manuscript does not contain any individual person's data in any form.

## Funding statement

No funding to be declared.

## Conflict of interest

The authors hereby declare that they have no conflict of interest.
